# Combined Overview on Diverticular Assessment:a new score for the management of diverticular disease

**DOI:** 10.1093/eurpub/ckac131.134

**Published:** 2022-10-25

**Authors:** G Nasi, A Tursi, F Di Mario, F Lammert, T Poskus, MC Reichert, J Regula, S Bonovas, M Sapienza, G Brandimarte

**Affiliations:** 1 Direction of Health Management, Cristo Re Hospital, Rome, Italy; 2 Territorial Gastroenterology Service, ASL BAT, Andria, Italy; 3 Gastroenterology, Maggiore Hospital, Parma, Italy; 4 Department of Medicine II, Saarland University Medical Center, Homburg, Germany; 5 Institute of Clinical Medicine, Vilnius University Hospital, Vilnius, Lithuania; 6 Gastroenterology, Medical Centre for Postgraduate Education, Warsaw, Poland; 7 Gastroenterology, Maria Sklodowska-Curie Memorial Cancer Centre and Institute of Oncology, Warsaw, Poland; 8 Department of Biomedical Sciences, Humanitas University, Rozzano, Italy; 9 Department of Life Sciences and Public Health, Catholic University, Rome, Italy; 10 Internal Medicine and Gastroenterology, Cristo Re Hospital, Rome, Italy

## Abstract

**Background:**

Diverticulosis is increasing worldwide as a public health problem. The Combined Overview on Diverticular Assessment (CODA) score, merging Diverticular Inflammation and Complication Assessment (DICA) and few clinical parameters, may reliably predict the occurrence of acute diverticulitis and surgery due to complications. Thus, the aim of the study is to confirm the value of DICA classification and to develop and validate the CODA endoscopic-clinical score.

**Methods:**

A number of 2198 patients, at the first diagnosis of diverticulosis/diverticular disease were enrolled in a multicentre, prospective, international cohort study. Participants were scored according to DICA classifications. A 3-year follow-up was performed. Survival methods for censored observation were used to develop and validate the CODA score for predicting diverticulitis and surgery.

**Results:**

The 3-year cumulative probability of diverticulitis and surgery was ≤4%, and ≤0.7% in CODA A; <10%and <2.5% in CODA B; >10%and >2.5% in CODA C, respectively. The 3-year cumulative probability of diverticulitis and surgery was of 3.3% (95% CI 2.5% to 4.5%) in DICA 1, 11.6% (95% CI 9.2% to 14.5%) in DICA 2 and 22.0% (95% CI 17.2% to 28.0%) in DICA 3 (p < 0.001), and 0.15% (95% CI 0.04% to 0.59%) in DICA 1, 3.0% (95% CI 1.9% to 4.7%) in DICA 2 and 11.0% (95% CI 7.5% to 16.0%) in DICA 3 (p < 0.001), respectively. The CODA score showed optimal discrimination capacity in predicting the risk of surgery in the development (cstatistic: 0.829; 95%CI 0.811 to 0.846) and validation cohort (c-statistic: 0.943; 95% CI 0.905 to 0.981).

**Conclusions:**

DICA endoscopic classification was confirmed to have a significant predictive value in terms of acute diverticulitis occurence/recurrence and risk of surgery. CODA score could provide a new risk stratification tool useful for everyday clinical practice and also with a significant public health impact in terms of treatment effectiveness and decision making.

**Key messages:**

• DICA endoscopic classification of diverticular disease is a clear predictor of the outcome of diverticulosis/diverticular disease.

• The CODA score, combining DICA and few clinical parameters, may reliably predict the occurence of acute diverticulitis and surgery due to complications.

